# Developmental iodine deficiency resulting in hypothyroidism reduces hippocampal ERK1/2 and CREB in lactational and adolescent rats

**DOI:** 10.1186/1471-2202-10-149

**Published:** 2009-12-18

**Authors:** Jing Dong, Wanyang Liu, Yi Wang, Yi Hou, Qi Xi, Jie Chen

**Affiliations:** 1 Department of Occupational and Environmental Health, School of Public Health, China Medical University, Shenyang, PR China; 2 Department of Physiology, Radboud University Nijmegen Medical Centre. Nijmegen, The Netherlands

## Abstract

**Background:**

Developmental iodine deficiency (ID) leads to inadequate thyroid hormone that impairs learning and memory with an unclear mechanism. Here, we show that hippocampal extracellular signal-regulated kinase (ERK1/2) and cAMP response element-binding protein (CREB) are implicated in the impaired learning and memory in lactational and adolescent rat hippocampus following developmental ID and hypothyroidism.

**Methods:**

Three developmental rat models were created by administrating dam rats with either iodine-deficient diet or propylthiouracil (PTU, 5 ppm or 15 ppm)-added drinking water from gestational day (GD) 6 till postnatal day (PN) 28. Then, the total and phorsporylated ERK1/2 and total and phorsporylated CREB in the hippocampus were detected with western blot on PN14, PN21, PN28 and PN42.

**Results:**

The iodine-deficient and hypothyroid pups showed lower serum FT_3 _and FT_4 _levels, smaller body size, and delayed eyes opening. The mean number of surviving cells in the hippocampus of the iodine-deficient and 15 ppm PTU-treated rats was significantly reduced compared to controls (P < 0.05). Iodine-deficient and 15 ppm PTU-treatment groups demonstrated significantly lower level of total and phosphorylated ERK1/2 and CREB than the controls on PN14, PN21 and PN28 (P < 0.05, respectively). The reduction of ERK1/2 and CREB was not reversible with the restoration of serum thyroid hormone concentrations on PN42.

**Conclusions:**

Developmental ID and hypothyroidism down-regulate hippocampal ERK1/2 and CREB in lactational and adolescent rats.

## Background

Iodine is an essential trace element and critical for the synthesis of triiodothyronine (T_3_) and thyroxine (T_4_). Thereby, iodine deficiency (ID) is the most common cause of hypothyroidism worldwide particularly in the developing countries [1]. In the process of early growth and development of most organs, hypothyroidism results in impaired brain development, cognitive deficits, impaired memory, impaired cognitive function and inattentiveness, and mental retardation in offspring [2-5]. During these developmental periods, the mother is the only source of iodine for the fetus [5-7]. Not until the day of birth is the offspring able to make sufficient levels of its own thyroid hormone. In line with this conclusion, recent researches show that children born to mothers experiencing subclinical perturbations of the thyroid axis during pregnancy have lower IQ scores and subtle deficits in cognition and memory [5,8,9]. Importantly, on one hand, ID induces irreversible developmental alterations in the fetuses and children central nervous system (CNS) [6]. On the other hand, ID is one of the greatest preventable cause of mental retardation and an important public-health problem [10]. This has been proven true in China where, after complying with Universal Salt Iodization (USI) in 1995, the ID disorders have been significantly reduced [11]. However, we recently reported that 7 of all 31 Chinese provinces or 400 counties with a population of 0.72 billion suffered from ID [11].

The hippocampus, a major component of the brain of humans and other mammals, belongs to the limbic system and plays important roles in long-term memory, learning processes and spatial navigation. The structural integrity of the hippocampus is dependent on sufficient thyroid hormone during development [8]. The classical genomic molecular mechanism of thyroid hormone is well understood [12]. After the uptake of T_3 _or T_4 _by target cells, T_3 _gains access to the cell nucleus and binds to nuclear thyroid hormone receptor (TR) [13,14]. In the nucleus, the facilitate binding of heterodimeric of TR and retinoic acid X receptor to thyroid hormone response elements regulates the consequent gene transcription through the action of co-repressors and co-activators [12,13]. Several lines of evidence imply that mitogen-activated protein kinases (MAPKs) mediate multiple cellular processes during normal brain development including gene expression, migration or trafficking, metabolism, differentiation, proliferation and apoptosis [15,16]. The MAPKs are also called extracellular signal-regulated kinases (ERK1 and ERK2) [16], which convey signals from cell surface receptors to the nucleus. This process is important in triggering the genomic response in neurons, and integrates signals from other transduction pathways [17]. It has been reported that ERK inhibition in the hippocampus led to disruption of spatial memory [18]. This is further supported by a recent study from Alzoubi and colleagues [19,20], showing that late long-term potentiation (LTP) depends on new protein synthesis through kinases-induced activation of cAMP-MAPK-CREB signaling pathway, leading to alteration of synaptic structure.

LTP is a well-accepted synaptic model of learning and memory [19,21] and thyroid hormone may play an indirect role in LTP by affecting MAPKs independent of nuclear thyroid receptors [16]. Firstly, thyroid hormone activates G-protein-coupled receptors, which activates ERK1/2, leading to CREB phosphorylation and cAMP response element (CRE) transcription [16,22,23]. It has been reported that MAPK/ERK activation is part of the non-genomic action of thyroid hormone [12]. MAPK signal transduction cascade is activated by T_4 _and a plasma membrane receptor on integrin αVβ3 via phospholipase C and protein kinase Cα. The activated MAPK can translocate to the nucleus to phosphorylate nuclear thyroid hormone receptor TRβ1, step de-repress TR and modulate intracellular protein trafficking of TR from cytoplasm to nucleus [12]. Furthermore, thyroid hormone has also been shown to regulate the expression and phosphorylation of ERK1/2 and CREB. Phosphorylation of ERK1/2 and CREB, in turn, causes important downstream effects and regulates the expression of a variety of proteins, such as immediate early genes, which are important in memory [24]. Therefore, it is not surprising that ERK1/2 and CREB play a critical role in LTP impairment following hypothyroidism [16,19,20,25].

However, little is know about how ID resulting in hypothyroidism regulates developmental hippocampus during lactational and adolescent period. It is widely accepted that neocorticogenesis begins at about embryonic day 13 (E13) and the postnatal development and maturation of the CNS persist for the lactation [from postnatal day (PN) 1 to weaning] and adolescence (from weaning to around PN 50) in rat [6]. So, transition from gestation to adolescent period is critical for CNS development and maturation. In adult rats, it has been shown that, thyroid hormones reduction by perchlorate irreversibly impairs synaptic transmission [26], where the restored thyroid hormone can not recover the developmental CNS impairments. In line with this study, our group has also previously shown in adult rats, developmental ID and hypothyroidism impairs LTP in CA1 region [27]. In contrast to many researches on adult animals exposed to developmental thyroid hormone insufficiency, there are very few experimental studies available to evaluate the alterations in early developmental period, following developmental ID and hypothyroidism. So far, it is still unclear whether ID and hypothyroidism have similar effects on ERK1/2, CREB and their phosphorylated fraction in hippocampal subregions in lactational and adolescent animals. In the present study, we shown that, in lactational and adolescent rats, hippocampal ERK1/2 and CREB are implicated in the brain impairment by developmental exposure to ID and 5 or 15 propylthiouracil (PTU).

## Methods

### Animals

Wistar rats (250-280 g) were obtained from the Center for Experimental Animals at China Medical University (Shenyang, China) with a National Animal Use License number of SCXK-LN 2003-0009. All experiments and surgical procedures were approved by the Animal Care and Use Committee at China Medical University, which complies with the National Institutes of Health Guide for the Care and Use of Laboratory Animals. All efforts were made to minimize the number of animals used and their suffering. Rats were housed under the following standard conditions temperature 24 ± 1°C and 12/12 h light/dark cycles food and water was provided *ad Libitum*. Animals were kept for 1 week before mating (♀:♂ = 2:1). The day of the vaginal plug was taken as gestational day (GD) 0. The pregnant rats were randomly assigned into four groups (n = 7 each): control group, 5 ppm PTU-treatment group, 15 ppm PTU-treatment group and iodine-deficient group. Control group received tap water and normal diet (iodine content: 470.50 ± 46.52 ng/g, measured by As^3+^-Ce^4+ ^catalytic spectrophotometry) during the experiment. Iodine-deficient group was administered with iodine-deficient diet (iodine content: 14.11 ± 1.96 ng/g) and tap water from GD6 till PN28. PTU-treated groups were administered 5 ppm and 15 ppm PTU (Sigma, St. Louis, MO) in the drinking water and fed with normal diet (iodine content: 470.50 ± 46.52 ng/g) from GD6 to PN28. The animal diet is made up of corn (46%), rice (40%), soybean (13%), calcium carbonate (0.5%), and sodium chloride (0.5%). For iodine-deficient diet, corn, rice, and soybean were obtained from the severe iodine-deficient area.

On GD21, the control group gave birth to 12-13 pups per litter. In contrast, 8-10 pups were born per litter in the treated groups. Each litter was culled to nine-ten pups on PN4 (same number of males and females in each group, if possible). Pups were weaned and each cage housed only two pups on PN25. Pup weights were recorded from PN3 to PN42. Eye opening was examined by daily observation between PN15 and PN20. The percent of pups per litter with both eyes open was calculated accordingly. Prior to the sacrifice on PN14, PN21 (lactation), PN28 and PN42 (adolescence), the same number of pups were randomly taken from different litters with a similar sex ratio in each group and weighed.

### Thyroid hormones

Thyroid hormone concentrations were determined via blood sampling collected from heart puncture of 8-10 pups in each group on PN14, PN21, PN28 and PN42. Briefly, after the pup was anesthetized using 99% ether and laid on its back, a 25-gauge needle attached to a 1 ml syringe was introduced at 10-30° from the horizontal axis of the sternum into the thoracic cavity to collect heart blood. Blood samples were centrifuged at 3,000 g for 5 min. Serum was then separated and stored at -70°C. All the serum was assayed by super-sensitive chemiluminescence immunoassay (IMMULITE, Diagnostic Products Corporation, Los Angeles, CA, USA) to measure thyroid stimulating hormone (TSH), free triiodothyronine (FT_3_) and free thyroxine (FT_4_). Hormones were measured in 300 μl aliquots. All samples were run in duplicate and the intra- and inter-assay variations were below 10%. The sensitivity of detection for TSH was 75 mIU/L, FT_3 _was 1.5-61 pmol/L, and FT_4 _was 3.9-77.2 pmol/L. Results below these limits of quantification were recorded as 75 mIU/L (TSH), 1.5 pmol/L (FT_3_) and 3.9 pmol/L (FT_4_), respectively for statistical purposes.

### Nissl staining

On PN14, PN21, PN28, and PN42, 5 pups in each group under deep anesthesia were intracardiac perfused with 50-100 ml normal saline containing 0.02% heparin followed by 200-400 ml 4% paraformaldehyde in 0.1 M potassium phosphate buffer (pH 7.4). The fixed brains were embedded in paraffin and sectioned into 6-μm-thick coronal sections on a microtome. Every third serial section was collected on gelatin-coated microscope slides. After deparaffinization in xylene for 10 min followed by 100% ethanol, the slides were washed in deionized water. Then, the slides were performed with routine Nissl staining based on the thionine technique and then analyzed under a microscope. The hippocampal subregions of interest were selected: CA1, CA3, and dentate gyrus (DG). All images were obtained under the same conditions of light illumination, at a magnification of 400×, with the microscope light source stabilized. For each group, quantitative data were acquired from the hippocampus on both sides of the brain. Cells with round and palely stained nuclei were considered to be surviving cells, whereas shrunken neurons with pyknotic nuclei were considered to be non-surviving cells. Every fifth brain section was selected from each animal and processed for cell counting in order to obtain an overall mean value for subsequent statistical analysis. Data were expressed as the number of surviving cells per field. The experimenter was blind to the experimental treatment of the individual animals during all data measurements.

### Western blot

On PN14, PN21, PN28 and PN42, 3 pups in each group, including males and females, were deeply anesthetized and euthanized by ether. Brains were removed and kept in an ice-cold artificial cerebrospinal fluid (ACSF) composed in miliMolar (mM): 124 mM NaCl, 3 mM KCl, 2 mM CaCl, 1 mM MgSO_4_, 1.25 mM NaH_2_PO_4_, 26 mM NaHCO_3_, and 10 mM glucose. According to the Paxions and Wastson atlas of the rat brain [28], the CA1, CA3 and DG regions of the hippocampus were immediately dissected out on ice and frozen at -70°C. Tissue samples were homogenized in 250 μl of buffered isotonic cocktail containing protease and phosphatase inhibitors (50 mM Tris-Hcl, 150 mM NaCl, 1% NP40, 0.1% SDS, 0.5% sodium deoxycholate, 10 mM NaF, 1 mM EGTA, 1 mM EDTA, and 0.2 mM PMSF). The samples were sonicated and incubated on ice for 30 min, and centrifuged at 13,000 g for 10 min at 4°C. The supernatants were centrifuged again and then removed. The total protein was estimated using coomassie brilliant blue assay. The samples were stored at -70°C until use.

Tissue lysates were diluted in sample buffer (0.312 mM Tris-HCl (pH 6.8), 50% glycerol, 10% SDS, 25% β-mercaptoethanol, and 0.25% bromophenol blue) to contain the same concentration of protein (3 μg/μl) and were then boiled at 100°C for 5 min. 10 μl aliquots of each sample (containing 30 μg protein) were loaded onto 10% SDS-acrylamide gels. Proteins were separated by the application of a constant voltage of 100 V for 1.5 h and then transferred onto nitrocellulose membranes at a constant voltage of 10 V for 45 min. After blocking the aspecific sites with PBS containing 0.1% Tween 20 (PBST) and 5% defatted dried milk, membranes were washed and incubated with rabbit anti-phospho-CREB monoclonal antibody (Cell Signaling Technology, Beverly, MA. 1:1000 dilution in PBS) for 2 h at room temperature. Rabbit polyclonal antibody for glyceraldehyde phosphodehydrogenase (GAPDH, Santa Cruz Biotechnology, America. 1:1000 dilution in PBS) was used as a loading control. The ratio of protein bands intensity to GAPDH band intensity was compared among the different groups. Membranes were washed and incubated in a horseradish peroxidase-conjugated anti-rabbit IgG (Beijing Zhongshan Goldenbridge Biotechnology Co., Ltd., China. 1:2500 dilution in PBS) for 1 h at room temperature before reaction with enhanced chemiluminescence (ECL) solution (Amersham, GE Healthcare, UK). Initial control experiments determined the optimal time of exposure to film, which was maintained throughout the experimental procedure. Membranes were exposed to film optimal time, and developed by hand. After developed and fixed, film was scanned. Protein bands were quantified with an image-analysis program (Gel Image System Ver. 4.00) and recorded with net optical density (NOD) corrected for background chemiluminescence. For all Western blots, on each gel GAPDH lanes were reserved for a quality control sample. The signals from target bands on a gel were normalized to the average signal for the quality control sample bands to simplify comparison across gels and reduce inter-gel variability.

Membranes were washed in restore Western blotting stripping buffer (Pierce Biotechnology, Inc., USA) for 15 min at room temperature after all the steps, and then washed in PBST. Following the operating instructions, we incubated the membrane with new SuperSignal West Working Solution and exposed it to film. If no signal was detected using a 5-min exposure, the HRP conjugate was successfully removed from the antigen or primary antibody. The membranes were stripped and re-incubated in rabbit anti-phospho-ERK1/2 monoclonal antibody, rabbit anti-CREB monoclonal antibody, and rabbit anti-ERK1/2 monoclonal antibody (all from Cell Signaling Technology, Beverly, MA; 1:1000 dilution in PBS) for 2 h at room temperature in turn. After incubation with primary antibodies, the membranes were washed and incubated in the horseradish peroxidase-conjugated anti-rabbit IgG (Beijing Zhongshan Goldenbridge Biotechnology Co., Ltd., China. 1:2500 dilution in PBS) for 1 h at room temperature before reaction with ECL solution respectively. Following the films were developed, fixed, and scanned, the protein band intensities were quantified with an image-analysis program.

### Statistics

Results were expressed as mean ± SEM. All analyses were carried out using the SPSS 11.5 software. For western blots, all of the values were normalized with GAPDH, which served as the internal control. The target band intensities in the treatment groups were normalized to those of the control group. The data differences among the multiple groups were analyzed using one-way analysis of variance (ANOVA). If *F *was significant, Tukey test was also used. *P*-values of less than 0.05 were considered statistically significant.

## Results

### Animal models

**Figure 1:**
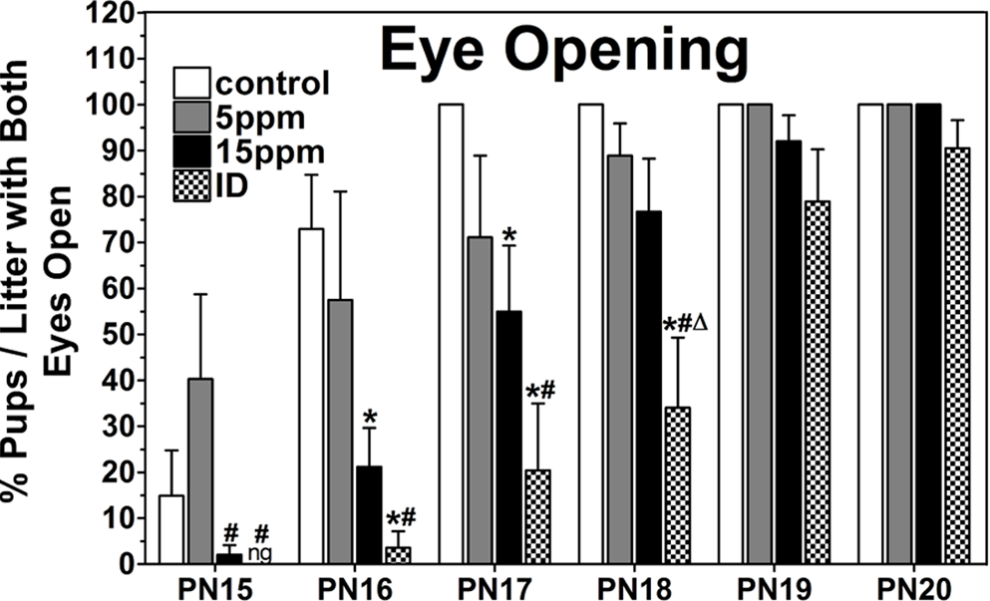
**ID and PTU-induced hypothyroidism delayed the eye opening in offspring**. The ratio of iodine-deficient and 15 ppm PTU-treatment pups within a litter with both eyes open was significantly lower than controls on PN17 and PN18. Each bar represents the mean ± SEM (control group: n = 6; 5 ppm treatment group: n = 6; 15 ppm treatment group: n = 6; iodine-deficient group: n = 7). *P < 0.05, as compared to control. ^#^P < 0.05, as compared to 5 ppm PTU-treatment group. ^Δ^P < 0.05, as compared to 15 ppm PTU-treatment group.

Numerous studies have shown that PTU treatment reduces offspring body weight [25,29,30]. Our data shown that the offspring's body weights in iodine-deficient, 15 ppm and 5 ppm PTU-treatment groups were statistically significant lower than the controls from PN3 to PN42 (P < 0.05, Table 1). Eye opening is a parameter that reflects the early physiological development of rats. Pups open their eyes normally between PN15 and PN19 [30]. ID and 15 ppm PTU-treatment delayed the pup eye opening to PN20. Further, comparing with the controls, the iodine-deficient and 15 ppm PTU-treated pups had a significantly lower eye opening percentage with both eye open per litter on PN17 and PN18 (P < 0.05, Fig. 1.). Dose-dependent reductions in thyroid hormones with concomitant elevations in TSH were observed in hypothyroid offspring by many researchers [25]. Interestingly and in line with this statement, our data showed that the offspring displayed hypothyroxinemia in iodine-deficient group (reduced FT_4 _with no significant reduction in TSH) and hypothyroidism in 15 ppm and 5 ppm PTU-treatment groups. TSH levels were increased significantly in 15 ppm and 5 ppm PTU-treatment offspring Vs controls (P < 0.05, Table 2.). The iodine-deficient and 15 ppm PTU-treatment groups had significantly lower serum FT_3 _and FT_4 _than the controls on PN14, PN21 and PN28 (P < 0.05, Table 2.). On PN42, the concentrations of serum FT_3_, FT_4 _and TSH in iodine-deficient and PTU-treatment groups were restored. Taken together, our results demonstrate that the iodine-deficient and 15 ppm PTU-treatment caused pup ID and hypothyroidism with developmental delay.

**Table 1:** ID and PTU-induced hypothyroidism reduced pups body weight (g)

Time	N	Control	5 ppm-PTU	15 ppm-PTU	ID
PN3	259	8.37 ± 0.12	7.06 ± 0.09 *	6.73 ± 0.11 *^#^	5.86 ± 0.08 *^#Δ^
PN7	252	13.79 ± 0.22	11.02 ± 0.17 *	10.66 ± 0.19 *	7.39 ± 0.15 *^#Δ^
PN10	231	20.13 ± 0.40	15.12 ± 0.16 *	14.29 ± 0.33 *	8.58 ± 0.22 *^#Δ^
PN14	217	26.43 ± 0.48	19.53 ± 0.24 *	18.09 ± 0.39 *^#^	10.43 ± 0.26 *^#Δ^
PN18	171	35.55 ± 0.59	25.96 ± 0.48 *	22.57 ± 0.53 *^#^	12.93 ± 0.46 *^#Δ^
PN21	140	44.83 ± 0.80	28.64 ± 0.73 *	25.05 ± 0.68 *^#^	14.41 ± 0.61 *^#Δ^
PN25	101	56.80 ± 1.39	38.97 ± 2.04 *	32.33 ± 1.27 *^#^	17.95 ± 1.04 *^#Δ^
PN28	101	74.22 ± 1.74	49.56 ± 1.97 *	37.14 ± 2.07 *^#^	21.41 ± 1.28 *^#Δ^
PN31	71	100.58 ± 2.52	69.31 ± 3.28 *	53.69 ± 4.27 *^#^	26.56 ± 1.69 *^#Δ^
PN35	71	126.30 ± 2.88	90.26 ± 3.85 *	72.17 ± 5.75 *^#^	40.94 ± 2.03 *^#Δ^
PN42	45	155.86 ± 6.11	127.09 ± 3.99 *	112.33 ± 7.40 *	69.41 ± 3.33 *^#Δ^

The pup weights of iodine-deficient, 15 ppm and 5 ppm PTU-treatment groups were significantly lower than controls from PN3 to PN42. Each values represents mean ± SEM. *P < 0.05, as compared to control group. ^#^P < 0.05, as compared to 5 ppm PTU-treatment group. ^Δ^*P *< 0.05, as compared to 15 ppm PTU-treatment group.

**Table 2:** Pup hormonal profile

Hormone	Time	Control	5 ppm-PTU	15 ppm-PTU	ID
TSH (mUI/L)	PN14	0.064 ± 0.029	1.713 ± 1.195 *	2.218 ± 0.587 *	0.142 ± 0.013 ^#Δ^
	PN21	0.052 ± 0.019	0.754 ± 0.538 *	1.990 ± 1.003 *^#^	0.246 ± 0.111 ^Δ^
	PN28	0.077 ± 0.023	0.908 ± 1.030 *	2.329 ± 1.378 *^#^	0.129 ± 0.038 ^Δ^
	PN42	0.074 ± 0.043	0.059 ± 0.031	0.065 ± 0.039	0.111 ± 0.049 *^#Δ^

FT3 (pmol/L)	PN14	3.093 ± 1.384	2.097 ± 0.401 *	1.540 ± 0.000 *	1.555 ± 0.021 *
	PN21	7.250 ± 1.205	9.823 ± 5.322	3.649 ± 1.229 *^#^	1.985 ± 0.346 *^#^
	PN28	13.310 ± 0.924	12.399 ± 2.823	6.879 ± 3.621 *^#^	6.095 ± 3.828 *^#^
	PN42	12.696 ± 4.882	13.105 ± 3.192	10.947 ± 3.282	13.520 ± 1.596

FT4 (pmol/L)	PN14	25.025 ± 6.060	7.997 ± 0.939 *	6.610 ± 0.935 *	8.850 ± 1.202 *
	PN21	33.225 ± 14.561	11.545 ± 6.173 *	10.000 ± 2.436 *	13.400 ± 0.707 *
	PN28	31.330 ± 6.527	10.968 ± 2.227 *	9.885 ± 2.184 *	12.430 ± 1.432 *
	PN42	41.155 ± 11.792	36.540 ± 7.826	41.150 ± 15.463	34.990 ± 9.212

The circulating levels of TSH were increased in PTU-induced hypothyroidism pups, while there were no significant differences in iodine-deficient pups on PN14, PN21 and PN28. ID and PTU-induced hypothyroidism decreased the serum FT_3 _and FT_4 _levels on PN14, PN21 and PN28. On PN42, the concentrations of serum FT_3_, FT_4 _and TSH in iodine-deficient and PTU-treatment groups were restored gradually. Each values represents mean ± SEM (n = 8-10 for each group). *P < 0.05, as compared to control group. ^#^*P *< 0.05, as compared to 5 ppm PTU-treatment group. ^Δ^*P *< 0.05, as compared to 15 ppm PTU-treatment group.

### ID and hypothyroidism increase hippocampal neuronal loss

To investigate whether or not hippocampal neuronal survival is impaired by ID and hypothyroidism, histological examination of hippocampal neurons was performed on Nissl-stained sections. The results reveal greater nuclear breakdown in the hippocampal neurons of offspring with low circulating thyroid hormone levels, in the CA1 (Additional file 1), CA3 (Additional file 2), and DG (Additional file 3) regions on PN14, PN21, PN28, and PN42. The mean number of surviving cells in the hippocampus of the iodine-deficient and 15 ppm PTU-treated rats was significantly reduced compared to controls (P < 0.05). These findings suggest that ID and hypothyroidism led to morphological damage in the hippocampus. Assessment of the simple effects of group showed that neuronal loss was increased at each time point in the hippocampus of rats exposed to the iodine-deficient or PTU-adulterated diet.

### ID and hypothyroidism reduce t-ERK1/2 and p-ERK1/2

**Figure 2:**
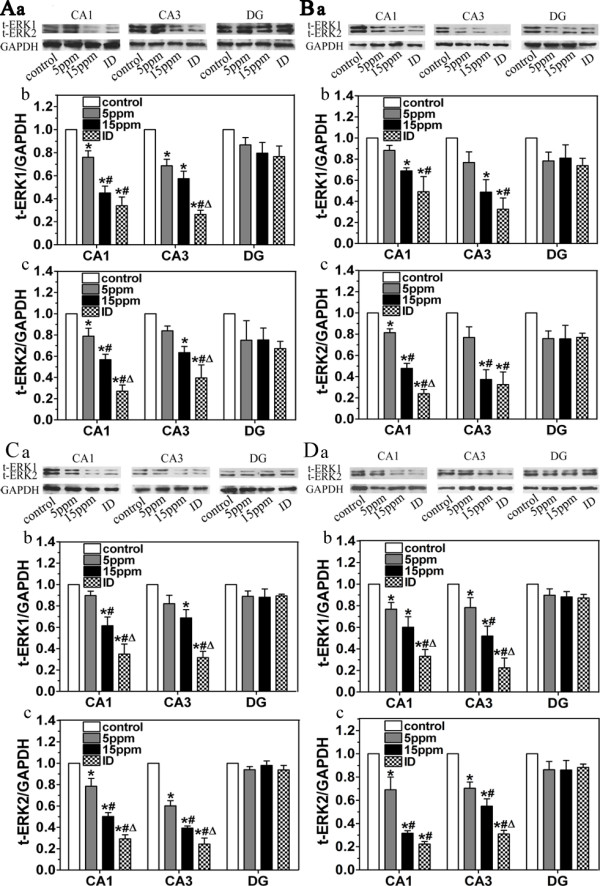
**Western blot analysis shows the decreased t-ERK1/2 following ID and hypothyroidism (n = 3 for each group)**. The levels of t-ERK1/2 in ID and hypothyroidism groups were significantly lower than controls in CA1 and CA3 regions on PN14 (A), PN21 (B), PN28 (C) and PN42 (D). (a) Representative blots of t-ERK1/2 in hipocampal CA1, CA3 and DG regions of control, 5 ppm PTU-treatment, 15 ppm PTU-treatment and iodine-deficient groups. (b and c) t-ERK1 and t-ERK2 levels. Each bar represents the mean ± SEM. *P < 0.05, as compared to control.^ #^*P *< 0.05, as compared to 5 ppm PTU-treatment group. ^Δ^*P *< 0.05, as compared to 15 ppm PTU-treatment group.

**Figure 3:**
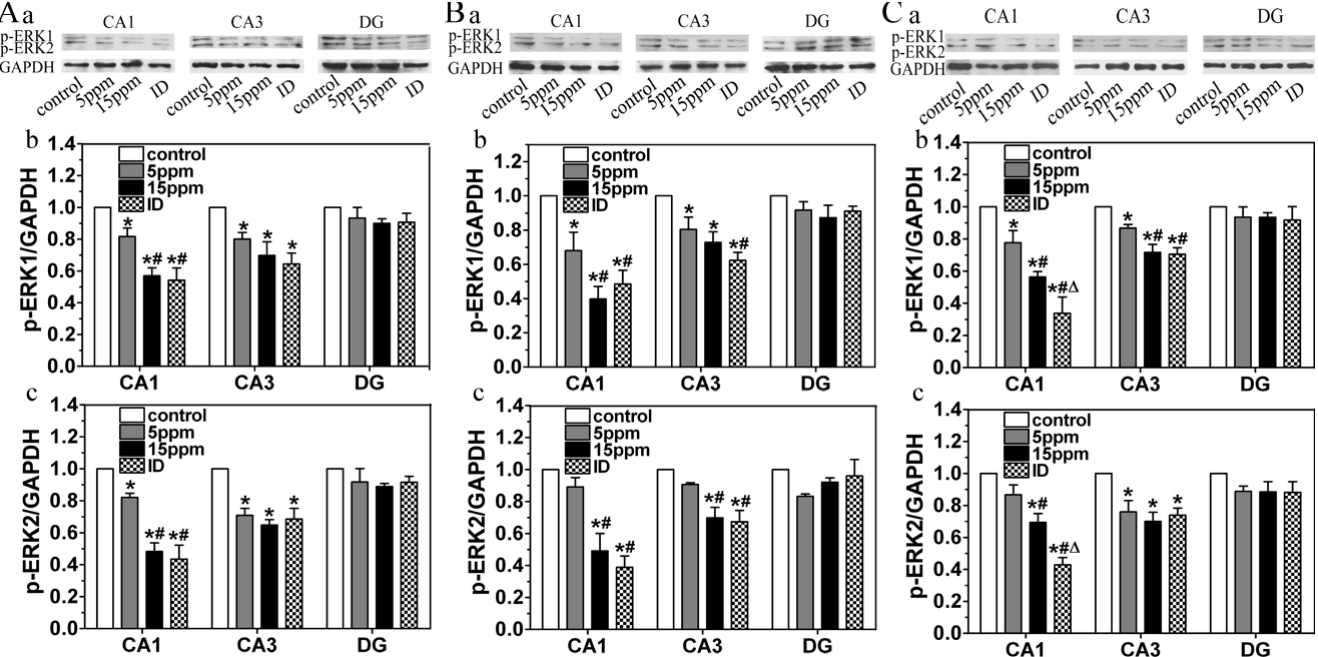
**Western blot analysis shows the decreased p-ERK1/2 following ID and hypothyroidism (n = 3 for each group)**. The levels of p-ERK1/2 in ID and hypothyroidism groups were significantly lower than controls in CA1 and CA3 regions on PN21 (A), PN28 (B) and PN42 (C). (a) Representative blots of p-ERK1/2 in hipocampal CA1, CA3 and DG regions of control, 5 ppm PTU-treatment, 15 ppm PTU-treatment and iodine-deficient groups. (b and c) p-ERK1 and p-ERK2 levels. Each bar represents the mean ± SEM. *P < 0.05, as compared to control. ^#^*P *< 0.05, as compared to 5 ppm PTU-treatment group. ^Δ^*P *< 0.05, as compared to 15 ppm PTU-treatment group.

Regulated by thyroid hormone and the role that they play in the hippocampus, ERK1/2 are important in the generation of learning and memory [16,19,20]. In the present study, we detected t-ERK1/2 and p-ERK1/2 changes in the pups following developmental ID and hypothyroidism using western blot approach. Both t-ERK1/2 and p-ERK1/2 were measured in CA1, CA3 and DG regions on PN14, PN21, PN28 and PN42 (Fig. 2 and Fig. 3, respectively). In CA1 and CA3 regions of the hippocampus, ID and hypothyroidism significantly reduced t-ERK1 (P44) or t-ERK2 (P42) (P < 0.05). p-ERK1 and p-ERK2 were significantly lower on PN21, PN28 and PN42 (P < 0.05). On the other hand, p-ERK1/2 was hardly detected on PN14. This might be due to lower t-ERK in early postnatal period in pups, and thus p-ERK1/2 signal becomes too weak to capture. In the DG region, however, ID and hypothyroidism did not change t-ERK1/2 or p-ERK1/2 expression.

### ID and hypothyroidism reduce t-CREB and p-CREB

**Figure 4:**
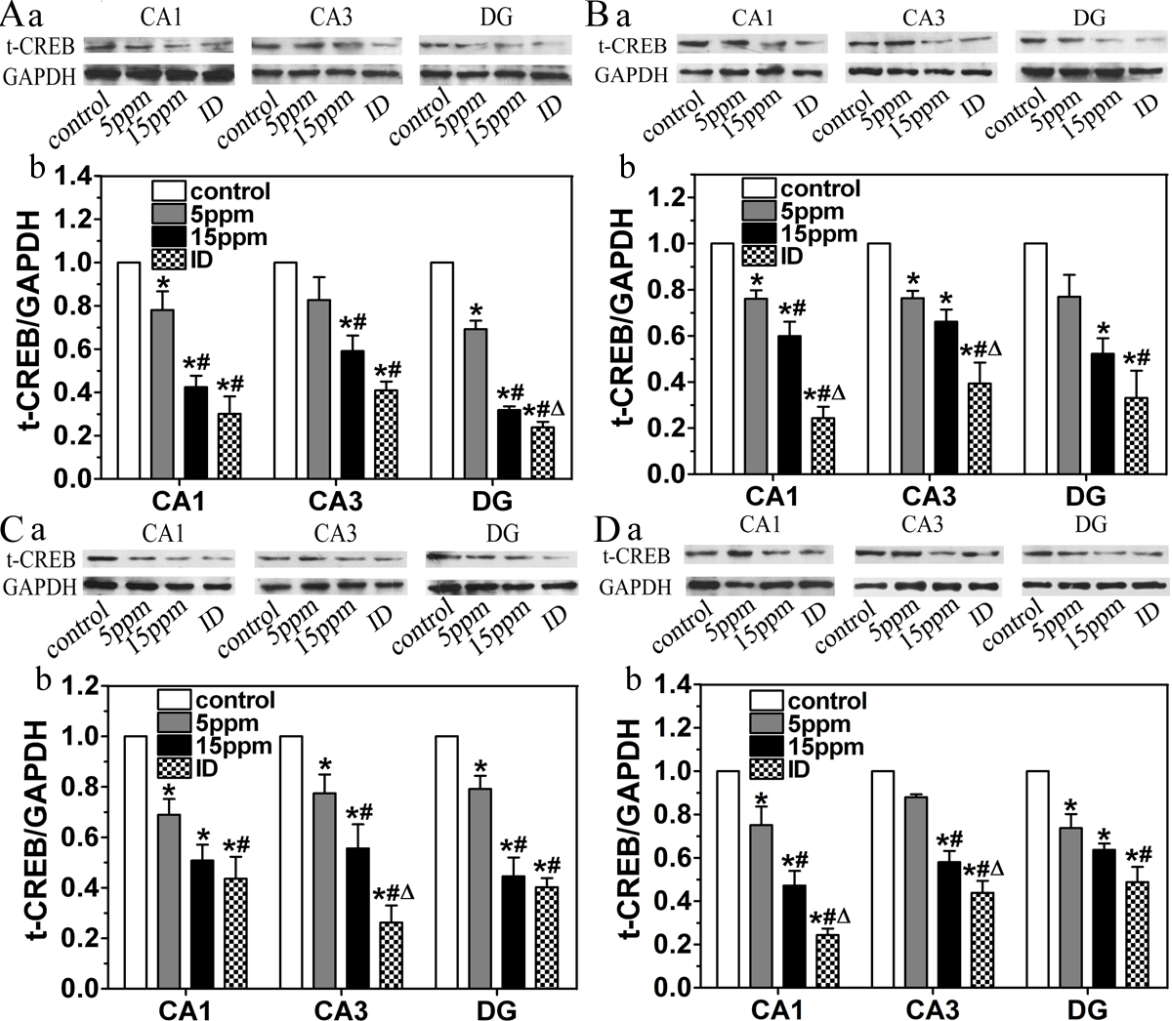
**Western blot analysis shows the decrease levels of t-CREB following ID and Hypothyroidism (n = 3 for each group)**. The levels of t-CREB in ID and hypothyroidism groups were significantly lower than controls on PN14 (A), PN21 (B), PN28 (C) and PN42 (D). (a) Representative blots of t- CREB in hipocampal CA1, CA3 and DG regions of control, 5 ppm PTU-treatment, 15 ppm PTU-treatment and iodine-deficient groups. (b) t- CREB levels. Each bar represents the mean ± SEM. *P < 0.05, as compared to control. ^#^*P *< 0.05, as compared to 5 ppm PTU-treatment group. ^Δ^*P *< 0.05, as compared to 15 ppm PTU-treatment group.

**Figure 5:**
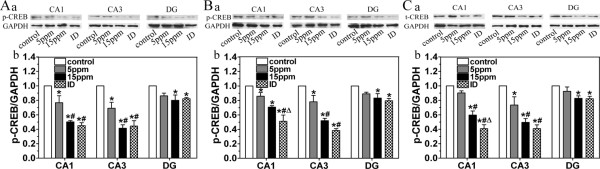
**Western blot analysis shows the decrease levels of p-CREB following ID and Hypothyroidism (n = 3 for each group)**. The levels of p-CREB in ID and hypothyroidism groups were significantly lower than controls on PN21 (A), PN28 (B) and PN42 (C). (a) Representative blots of p-CREB in hipocampal CA1, CA3 and DG regions of control, 5 ppm PTU-treatment, 15 ppm PTU-treatment and iodine-deficient groups. (b) p-CREB levels. Each bar represents the mean ± SEM. *P < 0.05, as compared to control.^ #^P < 0.05, as compared to 5 ppm PTU-treatment group. ^Δ^P < 0.05, as compared to 15 ppm PTU-treatment group.

As a downstream target molecule of ERK1/2, CREB plays a critical role in the generation of protein synthesis-dependent long-term changes in the brain [19] and is necessary for the fear-associated memory [31]. In order to investigate the effects of ID and hypothyroidism on CREB, t-CREB and p-CREB were detected via western blot. In the present study, t-CREB and p-CREB were clearly expressed in CA1, CA3 and DG regions on PN14, PN21, PN28 and PN42 (Fig. 4 and Fig. 5). However, the signals of p-CREB were very weak on PN14. ID and hypothyroidism significantly reduced both t-CREB (Fig. 4Ab) and p-CREB (Fig. 5Ab) in CA1, CA3 and DG regions (P < 0.05).

## Discussion

The major findings of this study are that, in lactation and adolescent stage of development rats, developmental ID and hypothyroidism (15 ppm PTU) significantly reduced the mean number of surviving cells in hippocampus and decreased ERK1/2 and CREB expression in hippocampal CA1 and CA3; even after the thyroid hormones back to normal, surviving cells, ERK1/2 and CREB were still lower than the controls. The present study demonstrates that developmental ID and hypothyroidism down-regulate hippocampal ERK1/2 and CREB in lactational and adolescent rats.

Our previous study has shown that ID was still a serious public health problem in China [32]. Given so many Chinese children exposed to developmental ID, this study sought to make three lactational and adolescent animal models to mimic the developmental exposure to ID and hypothyroidism. Many lines of literature using adult animal models have demonstrated that developmental hypothyroidism alters synaptic function in the hippocampus [16,19-21,25,27,33-35]. In agreement with these studies, the lactational and adolescent rats in this study have an irreversible impairment in hippocampal ERK1/2 and CREB following developmental exposure to ID and hypothyroidism. This is because that, during these developmental periods, the mother is the only source of iodine for the fetus and neonate [5-7,35,36]. Therefore, maternal ID leads to maternal and filial hypothyroxinemia. Taking together, developmental periods during pregnancy and lactation are critical in the brain development.

Thyroid hormone is well known to regulate morphological and biochemical alterations in brain during critical periods of brain development. This regulation can be done by genomic, posttranslational, and direct actions on neural substrates. Therefore, thyroid hormone is a fundamental factor that regulates normal development of the CNS. Logically, thyroid hormone insufficiency during early brain development is detrimental to synaptic transmission in hippocampus and to a cellular and behavioral model of learning and memory [2,5,8]. In need, our previous study has clearly demonstrated that ID-exposure lowers the children IQs in China [32]. Experimentally, we have also shown the ID can lead to an inhibition of LTP in the rat CA1 area [27].

It is well known that MAPK mediates neuronal metabolism, differentiation and proliferation [15,16]. Also, the persisting alteration in developmental brain involves changes in myelination and migration patterns of neurons [37-41]. The present study further confirmed the hypothesis that developmental ID and hypothyroidism induce irreversible impairment of two key proteins expression in the CA1 and CA3 regions, where both ERK1/2 and CREB expression was significantly reduced in either total protein level or phorsphorylated fraction. In agreement with our findings, Gilbert and colleagues observed the irreversibly CNS impairments in rats induced by developmental hypothyroidism from GD6 to PN30 [8,25,26,30]. This study provides us a clue to explore the mechanism by which thyroid hormone regulates brain development during lactational and adolescent periods.

As a member of MAPK, ERK1/2 is a critical memory-related protein in the generation of learning and memory. Thyroid hormone activates G-protein-coupled receptors, leads to ERK1/2 phosphorylation, and in turn enhances the transcription of some proteins that are important in memory [16]. It has been shown that hypothyroidism reduces the basal p-ERK1/2 in hippocampal CA1 region without affecting t-ERK1/2 [16,19,25]. In the DG region, however, hypothyroidism had no effect on t-ERK1/2 and p-ERK1/2 [16,19,25]. In the lactational and adolescent rats, here we show that t-ERK1/2 and p-ERK1/2 in iodine-deficient, 15 ppm PTU-treatment and 5 ppm PTU-treatment groups in CA1 and CA3 regions were significantly lower than controls; while there was no difference in DG region. This difference might be due to the different cell types of neurons in the different regions. In the DG region, the granule cells are more resistant to conditioned stimulus than the pyramidal cells [16]. This is in line with the importance of the CA1 and CA3 in the learning and memory generation. Recently, it is reported that MAPK genes are the direct targets of thyroid hormone [37,42] and MAPK activation is part of the non-genomic action of thyroid hormone [12]. Unlike the T_3_-dependent genomic mechanism, the non-genomic pathway involves T_4 _not T_3_. In present study, ID and hypothyroidism caused low T_4 _concentration in the lactational and adolescent rats. It is reported that in PTU treated pups, serum T_4 _concentration is negatively correlated with cerebrocortical type II 5'-deiodinase (D2) activity, a sensitive marker of tissue thyroid status and an indicator of brain's compensatory response to maintain cerebrocortical T_3 _[43]. Since T_3 _is correlated with synaptic response, in the present study, we speculate that the reduced serum FT_4 _may increase D2 activity, decrease cerebrocortical T_3 _concentration, and lead to bound-TR reduction. Taken together, ERK1/2 might be down-regulated by developmental ID and hypothyroidism via non-genomic pathway. Different from the present study, Calloni et al. reported that hypothyroidism promoted an increase in p-ERK1/2 [15]. It might be attributed to difference of the method on establishing hypothyroid animal model or the concentration of medicine. Calloni et al. administered pregnant rats with 0.02% methimazole (MMI) in drinking water from GD10 to the birth [15]. Since the neocorticogenesis and maturation of the CNS persist for the adolescence in rat [6], the early developmental impairments may be minor and reversible. Therefore, the increased p-ERK1/2 might be ascribed to the recovered morphological and physiological changes because of the termination of administration at birth.

Another key protein for the long-term memory processes is CREB. The activation of CREB and CREB-dependent transcriptional pathway is essential for memory consolidation [23,44]. Different from increased p-CREB in thyroid hormone treated neural cells [45], in the present study, developmental ID and hypothyroidism significantly lowered both t-CREB and p-CREB in CA1, CA3 and DG regions. It has been proposed that p-CREB decrease might attribute to protein reduction of t-CREB, Ca^2+^/Camodulin dependent protein kinase IV (CaMKIV), and adenylyl cyclase type I (ACI), or elevation of calcineurin [19,46]. In addition, TR activation can antagonize CREB-mediated transcription and inhibit the phosphorylation of CREB [47]. So far, the detailed mechanism by which developmental ID and hypothyroidism regulate CREB is still unclear. Since CREB is a downstream target molecule of ERK1/2, our present data imply that developmental ID and hypothyroidism may induce p-ERK1/2 reduction, which in turn lead to p-CREB reduction. However, we can not exclude the involvement of genomic pathway, where thyroid hormone modulates the CREB by regulating the transcriptions of CaMKIV.

Moreover, the number and morphology of neuron in hippocampus are affected by neonatal and adult hypothyroidism [48,49]. As a marker of neuronal development, Nissl body diminishes when neuronal impairment. We found here that the number of surviving neuronal cells in hippocampus in iodine-deficient and 15 ppm PTU-treatment groups were significantly lower than the controls. Importantly, the reduction of Nissle bodies in the developmental ID and hypothyroidism fits very well with the decreased protein production of ERK1/2 and CREB. This might be due to the decrease in cell number, and to a lower expression of the investigated genes in each neuron. Therefore, our observation on Nissl bodies further confirmed the irreversible CNS impairments following developmental ID and hypothyroidism.

In summary, this study has shed some light on one aspect of the ID/hypothyroidism-induced learning and memory impairment. The following questions are still unanswered: how does thyroid hormone regulate ERK and CREB? Why is there more reduction of p-CREB in DG region than p-ERK1/2? How do ERK1/2 and CREB regulate LTP production? Is ERK protein regulation due to a specific gene-expression modulation or are related to a general decrease in gene expression and/or protein synthesis following hypothyroidism? Further investigations are needed to answer these questions.

## Conclusion

Our previous study has confirmed the impairment of LTP induction following developmental ID and hypothyroidism [26]. The present study has further shown that, in lactational and adolescent rats, developmental ID and hypothyroidism induce irreversible reduction of ERK1/2 and CREB in hippocampal CA1 and CA3 regions. In conclusion, ERK1/2 and CREB may play an important role in ID and hypothyroidism-induced brain impairment in lactational and adolescent rats.

## Abbreviations

ACI: adenylyl cyclase type I; ACSF: artificial cerebrospinal fluid; ANOVA: analysis of variance; CaMKIV: Ca^2+^/Camodulin dependent protein kinase IV; CNS: central nervous system; CRE: cAMP response element; CREB: cAMP response element-binding protein; D2: type II 5'-deiodinase; DAB: 3, 30-diaminobenzidine; ECL: chemiluminescence; ERK: extracellular signal-regulated kinase;FT_3_: free triiodothyronine; FT_4_: free thyroxine; GAPDH: glyceraldehyde phosphodehydrogenase; GD: gestational day; HE: hematoxylin; ID: iodine deficiency; IEGs: immediate early genes; L-LTP: late long-term potentiation; LTP: long-term potentiation; MAPKs: mitogen-activated protein kinases; MEK: mitogen extracellular regulating kinase; MMI: methimazole; NMDA: N-methyl-D-asparticacid; NOD: optical density; PBS: phosphate-buffered saline; PN: postnatal day; PTU: propylthiouracil; SNK: Student-Newman-Keuls; T_3_: triiodothyronine; T_4_: thyroxine; TR: thyroid hormone receptor; TSH: thyroid stimulating hormone.

## Competing interests

The authors declare that they have no competing interests.

## Authors' contributions

JD, WL and JC conceived of the study, and participated in its design and coordination. JD drafted the manuscript. JD, YW and YH carried out the experiments, collected and analyzed the data. QX revised the manuscript. All authors participated in writing, and read and approved the final manuscript.

## Supplementary Material

Additional file 1 **ID and hypothyroidism induced neuronal loss in CA1 region (n = 5 for each group)**. Nissl staining was used to assess the numbers of surviving cells (round with palely stained nuclei) and dead cells (shrunken neurons with pyknotic nuclei) in the hippocampus. More dead pyramidal neurons were found in the CA1 region of the iodine-deficient and PTU-treated rats at PN21, PN28 and PN42 compared to controls. Representative photomicrographs are shown for each time point. Scale bar represents 25 μm, shown on lower left in (Aa), and applies to all panels. The Nissl-positive survival cell number of iodine-deficient and 15 ppm PTU-treatment groups was significantly lower than controls (B). Each value represents mean ± SEM. Significant differences from control group: *, P < 0.05. Click here for file

Additional file 2 **ID and hypothyroidism induced neuronal loss in CA3 region (n = 5 for each group)**. More dead pyramidal neurons were observed in the CA3 region of the iodine-deficient and 15 ppm PTU-treated rats on PN28 and PN42 compared to controls. Representative photomicrographs are shown for each time point. Scale bar represents 25 μm, shown on lower left in (Aa), and applies to all panels. The Nissl-positive survival cell number of iodine-deficient and 15 ppm PTU-treatment groups was significantly lower than controls (B). Each value represents mean ± SEM. Significant differences from control group: *, P < 0.05. Click here for file

Additional file 3 **ID and hypothyroidism induced neuronal loss in DG region (n = 5 for each group)**. More dead granular cells were found in the DG region of the iodine-deficient and 15 ppm PTU-treated rats on PN21, PN28, and PN42 compared to controls. Representative photomicrographs are shown for each time point. Scale bar represents 25 μm, shown on lower left in (Aa), and applies to all panels. The Nissl-positive survival cell number of iodine-deficient and 15 ppm PTU-treatment groups was significantly lower than controls (B). Each value represents mean ± SEM. Significant differences from control group: *, P < 0.05. Click here for file
