# Supplementary figures and images for: Developmental iodine deficiency resulting in hypothyroidism reduces hippocampal ERK1/2 and CREB in lactational and adolescent rats
Source: BMC Neurosci. 2009 Dec 18;10:149. doi: 10.1186/1471-2202-10-149 (PMC2804698; doi:10.1186/1471-2202-10-149)

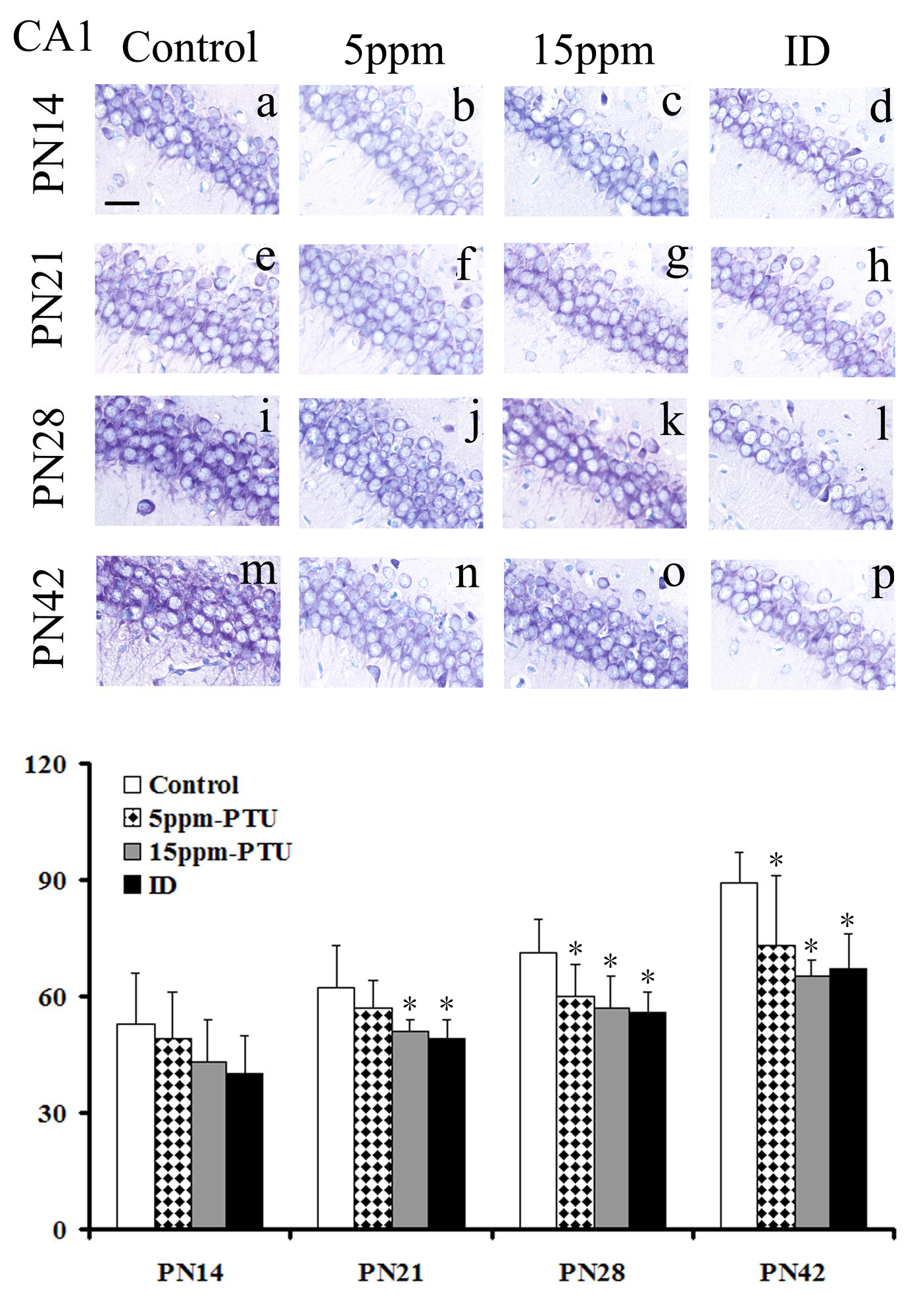

Supplement: Additional file 1 — ID and hypothyroidism induced neuronal loss in CA1 region (n = 5 for each group). Nissl staining was used to assess the numbers of surviving cells (round with palely stained nuclei) and dead cells (shrunken neurons with pyknotic nuclei) in the hippocampus. More dead pyramidal neurons were found in the CA1 region of the iodine-deficient and PTU-treated rats at PN21, PN28 and PN42 compared to controls. Representative photomicrographs are shown for each time point. Scale bar represents 25 μm, shown on lower left in (Aa), and applies to all panels. The Nissl-positive survival cell number of iodine-deficient and 15 ppm PTU-treatment groups was significantly lower than controls (B). Each value represents mean ± SEM. Significant differences from control group: *, P < 0.05. [file 1471-2202-10-149-S1.TIFF]

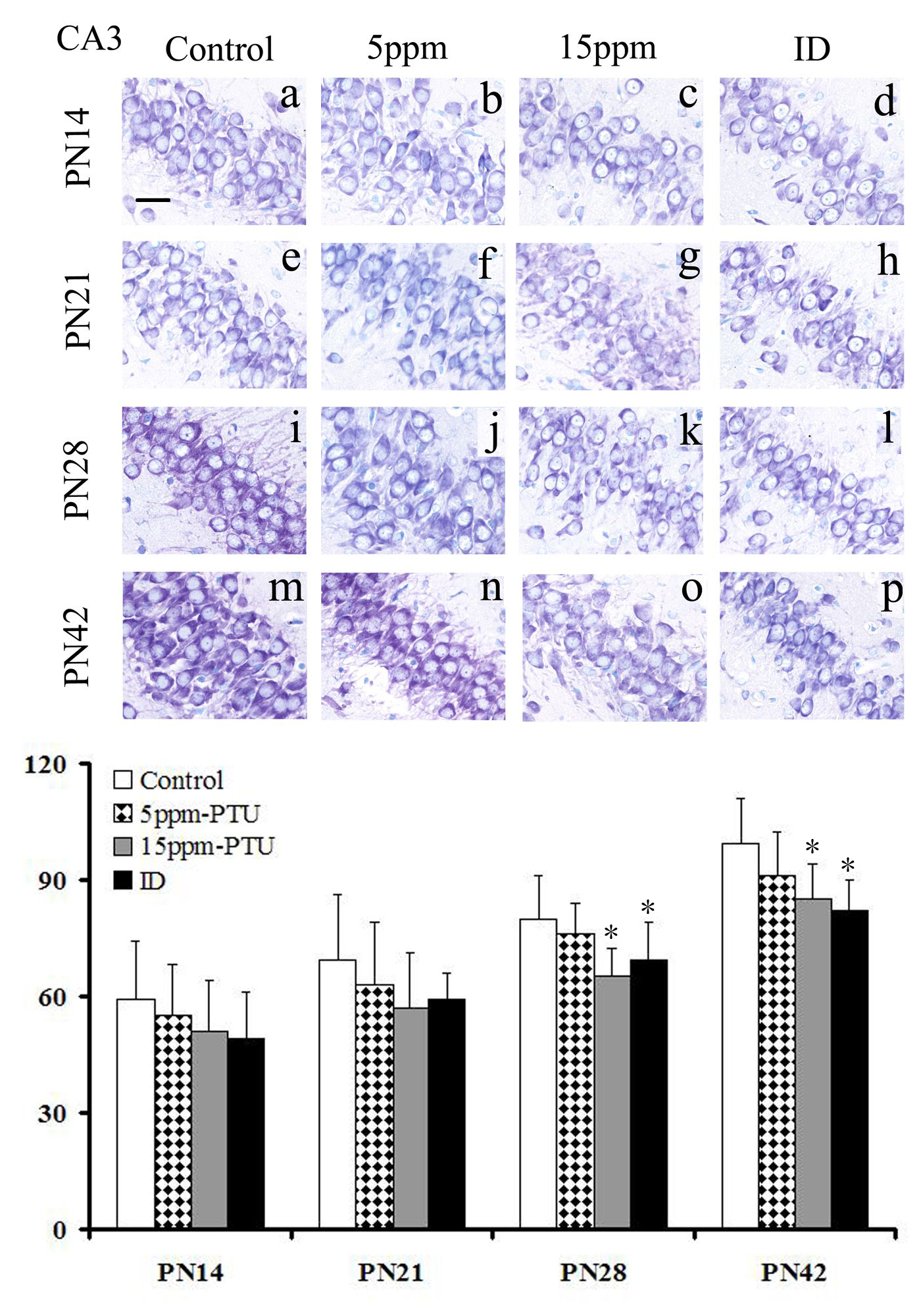

Supplement: Additional file 2 — ID and hypothyroidism induced neuronal loss in CA3 region (n = 5 for each group). More dead pyramidal neurons were observed in the CA3 region of the iodine-deficient and 15 ppm PTU-treated rats on PN28 and PN42 compared to controls. Representative photomicrographs are shown for each time point. Scale bar represents 25 μm, shown on lower left in (Aa), and applies to all panels. The Nissl-positive survival cell number of iodine-deficient and 15 ppm PTU-treatment groups was significantly lower than controls (B). Each value represents mean ± SEM. Significant differences from control group: *, P < 0.05. [file 1471-2202-10-149-S2.TIFF]

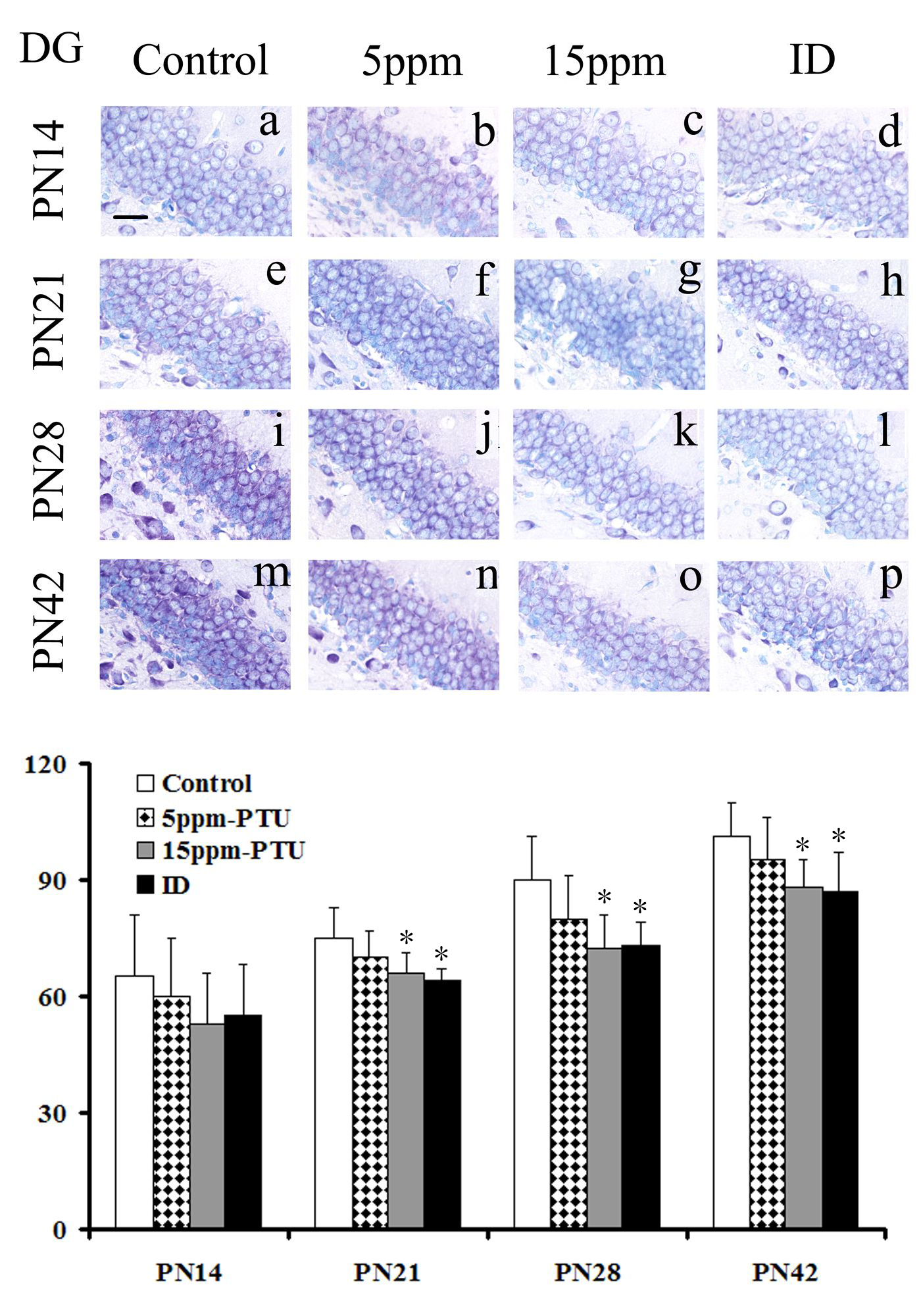

Supplement: Additional file 3 — ID and hypothyroidism induced neuronal loss in DG region (n = 5 for each group). More dead granular cells were found in the DG region of the iodine-deficient and 15 ppm PTU-treated rats on PN21, PN28, and PN42 compared to controls. Representative photomicrographs are shown for each time point. Scale bar represents 25 μm, shown on lower left in (Aa), and applies to all panels. The Nissl-positive survival cell number of iodine-deficient and 15 ppm PTU-treatment groups was significantly lower than controls (B). Each value represents mean ± SEM. Significant differences from control group: *, P < 0.05. [file 1471-2202-10-149-S3.TIFF]
